# Risk Factors for Unfavorable Treatment Outcomes among the Human Immunodeficiency Virus-Associated Tuberculosis Population in Tashkent City, Uzbekistan: 2013–2017

**DOI:** 10.3390/ijerph18094623

**Published:** 2021-04-27

**Authors:** Sherali Massavirov, Kristina Akopyan, Fazlkhan Abdugapparov, Ana Ciobanu, Arax Hovhanessyan, Mavluda Khodjaeva, Jamshid Gadoev, Nargiza Parpieva

**Affiliations:** 1 Department of Phthisiology and Pulmonology of the Tashkent Medical Academy, Tashkent 100109, Uzbekistan; fazlxan@mail.ru (F.A.); xodjaevam7724@gmail.com (M.K.); nargizaparpieva@gmail.com (N.P.); 2 WHO Regional Office for Europe, DK-2100 Copenhagen, Denmark; dr.akopian@gmail.com (K.A.); anna.ciobanu@gmail.com (A.C.); hovhannesyana@who.int (A.H.); 3 Tuberculosis Research and Prevention Center NGO, Yerevan 0070, Armenia; 4 World Health Organization Country Office in Uzbekistan, 16, Tarobiy Street, Tashkent 100100, Uzbekistan; gadoevj@who.int; 5 The Republican Specialized Scientific-Practical Medical Center of Phthisiology and Pulmonology, Tashkent 100086, Uzbekistan

**Keywords:** tuberculosis, treatment outcomes, HIV, opportunistic infection, diabetes, hepatitis C, Uzbekistan, central Asia, operational research, SORT IT

## Abstract

Tuberculosis (TB) and human immunodeficiency virus (HIV) co-infection poses a growing clinical challenge. People living with HIV have a higher chance of developing TB, and once the disease has progressed, are at greater risk of having unfavorable TB treatment outcomes. Data on TB treatment outcomes among the HIV-associated TB population in Uzbekistan are limited. Thus, we conducted a cohort study among 808 adult patients with HIV-associated TB registered at the Tashkent TB referral hospital from 2013–2017 to document baseline characteristics and evaluate risk factors for unfavorable TB treatment outcomes. The data were collected from medical records and ambulatory cards. About 79.8% of the study population had favorable treatment outcomes. Antiretroviral therapy (ART) coverage at the admission was 26.9%. Information on CD4-cell counts and viral loads were largely missing. Having extrapulmonary TB (aOR 2.21, 95% CI: 1.38–3.53, *p* = 0.001), positive sputum smear laboratory results on admission (aOR 1.62, 95% CI: 1.07–2.40), diabetes (aOR 5.16, 95% CI: 1.77–14.98), and hepatitis C (aOR 1.68, 95% CI: 1.14–2.46) were independent risk factors for developing unfavorable TB treatment outcomes. The study findings provide evidence for targeted clinical management in co-infected patients with risk factors. Strengthening the integration of TB/HIV services may improve availability of key data to improve co-infection management.

## 1. Introduction

Tuberculosis (TB) and human immunodeficiency virus (HIV) co-infection remain a major problem in clinical practice, greatly contributing to the burden of infectious diseases globally [1]. In 2019, an estimated 10 million people fell ill with TB, of whom 8.2% were people living with HIV (PLHIV) [2]. TB remains the leading cause of death among PLHIV [2]. In 2019, it was estimated by the World Health Organization (WHO) that globally 208,000 (range 177,000–242,000) HIV-positive people died from TB, which constitutes about one third of all HIV deaths [2]. In 2019, the actual number of reported TB cases was 456,426 among PLHIV; this is about 56% of the estimated burden, meaning that only around half of the estimated patients were detected [2]. In 2019, antiretroviral therapy (ART) coverage among all PLHIV comprised only 67% globally [2]. Systematic screening of TB among the HIV population is recommended by WHO to assure early diagnosis [3].

The high incidence of TB in PLHIV is explained by two main reasons: increased susceptibility to infection with *Mycobacterium tuberculosis* and high risk of reactivation of latent TB infection [4]. The decline, destruction, and dysfunction of CD4 T-lymphocyte cells and the impairment of macrophage function in PLHIV weaken the ability of the immune system to contain TB [5]. This increases the risk of development of the disease, either by primary TB infection or by reactivation of latent TB infection. Studies from 2018 show that HIV increases the risk of TB by about 19 times (range 15–22) [6]. Among untreated PLHIV, the risk of reactivation of latent TB infection is 3–16% annually, which is almost the same as the lifetime risk of TB in people without HIV (5–10%) [7]. In the first year after HIV infection, the risk of TB doubles and then keeps rising as immunodeficiency progresses [8]. Conversely, TB augments HIV viral replication and viral growth, both of which negatively affect the course of HIV infection [9,10]. Thus, both TB and HIV interfere and negatively affect each other and the course of co-infection.

HIV also negatively influences TB treatment outcomes in a variety of different ways [2]. The HIV-associated TB population receives antiretroviral therapy (ART), TB treatment, and prophylactic treatment for other opportunistic infections. Thus, they are more prone to adverse drug reactions and, as a result, treatment regimen interruption. The high medication pill burden can lead to poor treatment adherence [11]. Not receiving ART, having a CD4-cell count <200 cells/µL, being in WHO HIV clinical stage 4, having other comorbidities, being older, and not receiving co-trimoxazole prophylaxis are some of the major risk factors for unfavorable TB treatment outcomes among patients with HIV associated TB [11,12,13].

In recent decades, Uzbekistan has made major improvements in the delivery of care for the general TB population, as well as for those with HIV-associated TB. Among the key achievements are ART scale up at the national level (ART coverage increased from 19% in 2010 to 58% in 2019), an increase in ART coverage in the HIV-associated TB population (from 36.8% in 2010 to 83.2% in 2018), a significant decrease in the number of reported TB cases (from 94 per 100,000 in 2003 to 49 per 100,000 population in 2019), and the adoption of national protocols for managing TB and HIV based on WHO-recommended guidelines [2,14,15]. Despite these achievements, the country faces challenges related to drug resistant TB, and ART coverage is still below the global average [2].

According to the WHO, in 2017 in Uzbekistan, the number of patients with positive HIV status comprised 5.6% (935) of new and relapsed TB patients [16] and this ranged from 3.5% to 6.4% during the 2013–2017 period [17]. During the same period, ART coverage in the HIV-associated TB population ranged from 45.4% to 100% [17]. In 2017, the ART coverage in the HIV-associated TB population was 100%, but among all HIV positive patients it was only 51% [16,18]. According to the national protocol and guidelines for managing HIV infection, it is recommended that all PLHIV undergo regular TB screening. Similarly, all TB patients should be tested for HIV [14]. By geographical distribution, the prevalence of HIV infection is higher in the large cities of the republic and in areas where the population density is higher. About 40% of reported cases reside in the city of Tashkent and the Tashkent region (the capital and the surrounding region) [19].

Despite the high burden of TB/HIV co-infection in the country, we have not been able to identify any formal research that has assessed risk factors for unfavorable TB treatment outcomes among the HIV-associated TB population in Uzbekistan. Thus, we conducted a study to address this knowledge gap and generate evidence and recommendations for targeted practice and programmatic management. Our study aimed to document baseline characteristics and treatment outcomes and assess risk factors for unfavorable TB treatment outcomes among patients with HIV-associated TB registered in Tashkent city, Uzbekistan, from 2013–2017.

## 2. Materials and Methods

### 2.1. Study Design

This was a cohort study using routine secondary data.

### 2.2. Setting

#### 2.2.1. General Setting

Uzbekistan is a low middle-income country located in Central Asia, with a population of around 33 million population. It consists of twelve regions, an autonomous republic, and a capital city Tashkent with about 2.5 million residents.

#### 2.2.2. Specific Setting

The Republican Specialized Scientific-Practical Medical Center of Phthisiology and Pulmonology (RSSPMCPP) manages and implements a broad spectrum of TB control activities within the country and operates as the National TB Program (NTP) of the republic. All patients receive TB treatment free of charge in accordance with WHO treatment recommendations. Private clinics do not provide any TB treatment. The City Clinical Hospital of Phthisiology and Pulmonology (CCHPP) is in charge of the provision of specialized medical care to all patients in Tashkent city who have a confirmed diagnosis of TB/HIV co-infection. The HIV-associated TB population is referred to the CCHPP from five inter-district phthisiatric dispensaries in Tashkent.

There is a National Clinical Guideline for TB and HIV coinfection diagnosis and management, which is based on the WHO key recommendations, including screening of HIV patients for TB and administration of isoniazid preventive therapy [14]. PLHIV are screened for clinical signs of TB, and undergo X-ray assessment and rapid diagnostic evaluation with GeneXpert MTB/RIF. In co-infected patients, TB treatment is initiated as soon as active TB disease has been diagnosed. ART is initiated within 2 weeks after the start of the TB treatment for patients with CD4-cell count <50 cells/mm^3^, and within 2–8 weeks for patients with CD4-cell count >50 cells/mm^3^, taking into consideration drug-drug interactions and how well the TB treatment is tolerated. TB treatment outcomes are standardized and based on WHO guidelines [14].

### 2.3. Study Population

All adults (>18 years old) with HIV-associated TB registered in the CCHPP from 2013 to 2017 were included in the study. No exclusion criteria were applied.

### 2.4. Data Variables and Sources

Medical records of patients registered in the CCHPP during the period 2013 to 2017 served as the main data source. Data on treatment outcomes were obtained from the medical cards of each patient, which were available in the respective ambulatories of Tashkent.

The main study outcome variable was TB treatment outcome. We categorized the TB treatment outcome variable into dichotomous variables denoting favorable (combined cured and treatment completed) and unfavorable (combined failure, death, and lost to follow-up) outcomes.

The study independent variables included: socio-demographic and behavioral characteristics (age, sex, body mass index (BMI), employment and marital status, educational level, self-reported current smoking status, and confirmed drug abuse and alcohol abuse); TB characteristics (TB treatment history, site of disease, rifampicin-resistance (results of drug susceptibility testing), results of sputum smear microscopy on admission, bacteriological confirmation of the disease, the treatment regimen at the initiation of treatment, and complications of clinical diagnosis); HIV characteristics (ART at the time of admission, CD4-cell count, and viral load); and comorbidities such as hepatitis C, mycotic infection (the presence of laboratory confirmed mycotic infection in sputum or smear test), and diabetes mellitus (patients who have a prior diagnosis and are registered at the ambulatory level). In addition, we extracted information on whether fluconazole preventive treatment was administered.

### 2.5. Analysis and Statistics

A data documentation sheet was used to enter the data. Data were cleaned through random checks, range, and spot checking. Data were analyzed using Intercooled Stata software version 15 (Stata Corp. College Station, TX, USA). We summarized socio-demographic and clinical characteristics of the study population with frequencies and proportions (for categorical variables), and means, standard deviations, and interquartile ranges (for continuous variables) as appropriate.

Unadjusted analysis was done using simple logistic regression for each potential independent variable. Variables significant at *p* < 0.1.0 and key variables (age, sex, and receiving ART on admission) were included in the final model. Forward (step-up) selection was used to build the final regression model. The level of significance was set at *p* < 0.05. Estimates with 95% confidence intervals (CI) were presented in respective tables.

## 3. Results

Overall, 828 patients with HIV-associated TB were registered in the clinic during the study period. Information was available for 808 patients and all of these patients were included in the study. The mean age of the study population was 41.2 years (SD: ±8.3) and majority of patients were males (626/808, 77.5%). Almost one third of participants were underweight with a BMI below 18.5 kg/m^2^ (220/808, 28.3%). More than half of the participants were smokers (490/783, 62.6%). Socio-demographic characteristics of the study population are presented in Table 1.

### 3.1. TB and HIV Characteristics

Clinical characteristics of the study population are presented in Table 2. The majority of patients were newly registered (670/806, 83.1%), over three quarters had bacteriological confirmation of the disease (623/808, 77.1%), and just under three quarters were on treatment including first-line drugs (585/803, 72.9%). Amongst those for whom data was available, rifampicin-resistance was found in 29.4% (208/707). Favorable treatment outcomes were registered in 79.8% patients (644/807)—cured (61.0%) and completed (18.8%). Unfavorable treatment outcomes included failure (1.4%), loss to follow-up (7.9%), death (9.2%), and not evaluated (1.7%).

Date of HIV registration was available for 90% (731/808) of patients. About 93.8% of patients were diagnosed with HIV two months prior to the initiation of TB treatment (median: 72.3 months, IQR: 23.8–117, range: (−0.5) −243.2). Only 81.4% (658/808) of the cohort had recorded information about ART at the time of admission, and of those 26.9% (177/658) were on ART. As for CD4-cell count, only 18.4% (149/808) of the cohort had recorded information. The mean CD4-cell count per µL was 204 (SD: ±160.2, IQR: 76–300, range: 2–847). Viral load information was missing for 99.7% of the cohort.

Diabetes was recorded in 2% (15/808) and hepatitis C in about 30% (239/802) of the cohort. Information on mycotic infection of the respiratory tract was available only for 465 patients, and of these 44.3% had mycotic infection. Of those for whom data were available, 13.5% (103/765) received fluconazole preventive therapy.

### 3.2. Risk Factors for TB Treatment Outcomes

Unadjusted analysis showed that patients with a history of previous TB treatment, extrapulmonary TB, a positive sputum smear examination, hepatitis C, and diabetes had statistically significantly higher odds of developing unfavorable TB treatment outcomes. Patients with bacteriological confirmation of TB had lower odds of developing unfavorable TB treatment outcomes (Table 1 and Table 2).

Adjusted analysis showed that having extrapulmonary TB, positive sputum smear laboratory results, diabetes and hepatitis C were independent risk factors for developing unfavorable TB treatment outcomes. Patients with extrapulmonary TB (in comparison to patients with pulmonary TB) had 2.21-times higher odds of developing unfavorable treatment outcomes (aOR 2.21, 95% CI: 1.38–3.53). Patients who had positive sputum smear results (in comparison to patients with negative sputum smear results) had 62% higher odds of unfavorable TB treatment outcomes (aOR 1.62, 95% CI: 1.07–2.40). Having hepatitis C was associated with 68% higher odds (aOR 1.68, 95% CI: 1.14–2.46) and having diabetes was associated with 5-times higher odds (aOR 5.16, 95% CI: 1.77–14.98) of developing unfavorable TB outcomes (Table 3).

## 4. Discussion

This study is the first report from Uzbekistan about risk factors for unfavorable treatment outcomes among patients with HIV-associated TB.

The study found that a high proportion (79.8%) of patients with HIV-associated TB in Tashkent city, Uzbekistan, had favorable treatment outcomes. The proportion is higher than that reported in the WHO European region (51% in 2018) and globally (76% in 2018) [2]. Furthermore, the proportions with favorable treatment outcomes were similar among patients with and without rifampicin resistance. Our findings align with those reported from Thailand where a high treatment success rate (89.8%) was documented among HIV-associated TB patients [20]. However, it is widely known and confirmed in many publications and surveillance reports that treatment outcomes worsen in patients with resistance profiles. Our encouraging results from Uzbekistan need to be confirmed through further research conducted on larger numbers of patients and with more complete data recording and reporting.

We found that in our cohort, extrapulmonary TB, positive sputum smear results on admission, and comorbidities such as diabetes and hepatitis C were independent risk factors for unfavorable treatment outcomes.

Extrapulmonary TB was associated with unfavorable treatment outcomes in our study. Our findings are in line with the previous reports. The published literature shows that extrapulmonary TB is more common in the HIV-associated TB population, it becomes increasingly prevalent with progressive immunodeficiency, and it contributes to unfavorable TB treatment outcomes [21,22,23,24,25]. A study conducted in sub-Saharan Africa showed that extrapulmonary TB was associated with an increased risk of unfavorable outcomes such as death and lost to follow-up among patients with HIV-associated TB [21].

Diabetes is a known risk factor for unfavorable TB treatment outcomes, and our findings align with the published literature. The prevalence of diabetes in our cohort was lower than in other settings, although we were not able to identify literature on the prevalence of diabetes and impact on treatment outcomes specifically for the HIV-TB population. A study conducted in Armenia showed that the prevalence of diabetes among the general TB population was 5.8%, and diabetes was an independent risk factor for unfavorable treatment outcomes (aOR = 8.99) [26]. A large prospective cohort study conducted in Mexico among the TB population showed that having diabetes increased the risk of unfavorable outcomes by 2.93 times and the prevalence of diabetes in this Mexico study was 29.6% [27]. In another study conducted in India, the prevalence of diabetes among TB patients was 25.3% [28] and a pooled prevalence of diabetes among the TB population in Africa was 9.0% [29]. In line with our findings, it is important to pay greater attention to the screening of diabetes among the HIV-associated TB population in Uzbekistan.

Hepatis C has been associated with unfavorable treatment outcomes in the literature and is known to worsen the clinical management of HIV-associated TB. Two previous studies have explored the presence of hepatitis C coinfection in relation to unfavorable treatment outcomes among HIV-associated TB patients [30,31]. Both studies were conducted in Thailand. One study found that the odds of death were higher among anti-hepatitis C virus (HCV) reactive patients compared to patients who were non-reactive to any other viral marker [31]. The other study found that patients with hepatitis C antibody reactivity had a higher risk of death [30]. Our study only used anti-HCV antibody testing (which was confirmed by a second test) and did not include other hepatitis C laboratory markers; nevertheless, our key findings mirror those reported in other published studies.

ART coverage in our cohort was low (26.9%), compared to national statistics of ART coverage among HIV-associated TB patients from 2013–2017 (45.4–100%). This discrepancy might be due to the large amount of missing information on ART in our cohort. In addition, those recorded as not being on ART at the time of admission might have interrupted their treatment at that specific time. In our cohort, there was a high proportion of missing information on CD4-cell count, viral load, and ART which are crucial indicators in the management of HIV-associated TB. Further research where there is more complete reporting of HIV data to TB facilities could shed light on the issue. Strengthening the integration of HIV-TB services may also ease the information flow with respect to management and surveillance of HIV-TB co-infection.

A big strength of the study is that the large sample size allowed us to identify independent risk factors for unfavorable treatment outcomes. In addition, the study findings are generalizable to the largest city in Uzbekistan that accounts for a large proportion of patients with HIV-associated TB in the country (40%) [19].

The study has several limitations. First, some of the collected variables might have introduced bias to our findings. Second, the data on treatment regimens were available only for the initiation of treatment and did not reflect changes of treatment that might have occurred after drug-susceptibility testing results were available. Thus, the data on treatment regimens might be incorrect. Third, because of large numbers of missing values, we were not able to adjust for CD4-cell count and viral load, which are known risk factors for unfavorable treatment outcomes among HIV-associated TB patients. Fourth, the data on hepatitis C and diabetes treatment and detailed resistance profile were not available to evaluate their impact on the TB treatment outcomes.

## 5. Conclusions

This study showed that in Tashkent city during the period 2013–2017, a high proportion (79.8%) of the HIV-associated TB population had favorable treatment outcomes, which is higher than in the WHO European region. ART coverage was lower compared to national statistics. We found that having extrapulmonary TB, positive sputum smear results on admission, diabetes, and hepatitis C were independent risk factors for unfavorable treatment outcomes.

## Figures and Tables

**Table 1:** Socio-demographic characteristics among human immunodeficiency virus (HIV)-associated tuberculosis (TB) patients registered in Tashkent city, Uzbekistan (2013–2017) and their association with TB treatment outcomes.

Characteristics	Total		Favorable Treatment Outcome		Unfavorable Treatment Outcome		Unadjusted OR	95%CI	*p*-Value
	N	(c%)	N	(r%)	N	(r%)			
Age									
below 30 years	58	(7.2)	47	(81.0)	11	(19.0)	**1**		
30–39 years	295	(36.5)	236	(80.0)	59	(20.0)	1.06	(0.52–2.19)	0.857
40–49 years	334	(41.3)	267	(79.9)	66	(19.8)	1.05	(0.52–2.15)	0.880
50 years and over	121	(15.0)	94	(77.7)	27	(22.3)	1.22	(0.56–2.60)	0.609
Gender									
Male	626	(77.5)	494	(78.9)	131	(20.9)	1		
Female	182	(22.5)	150	(82.4)	32	(17.6)	0.81	(0.52–1.23)	0.319
Body mass-index (31 missing)									
≥18.5 kg/m^2^	557	(71.7)	454	(81.5)	102	(18.3)	1		
<18.5 kg/m^2^	220	(28.3)	174	(79.1)	46	(20.9)	1.18	(0.80–1.74)	0.413
Current smoking status (26 missing)									
No	292	(37.3)	234	(80.1)	59	(20.2)	1		
Yes	490	(62.6)	393	(80.2)	97	(19.8)	0.97	(0.68–1.41)	0.908
Alcohol abuse									
No	427	(52.8)	345	(80.8)	81	(19.0)	1		
Yes	381	(47.2)	299	(78.5)	82	(21.5)	1.16	(0.83–1.65)	0.374
Drug abuse (43 missing)									
No	574	(75.0)	458	(79.8)	116	(20.2)	1		
Yes	191	(25.0)	153	(80.1)	38	(19.9)	1.02	(0.67–1.54)	0.925
Employment status (4 missing)									
Yes	68	(8.5)	56	(82.4)	12	(17.6)	1		
No	736	(91.5)	587	(79.8)	148	(20.1)	1.17	(0.61–2.25)	0.623
Educational level (14 missing)									
No education	13	(1.6)	13	(100.0)	0	(0.0)			
Secondary	746	(94.0)	596	(79.9)	149	(20.1)	1		
Special	14	(1.8)	11	(78.6)	3	(21.4)	1.09	(0.30–3.96)	0.895
Higher	21	(2.6)	17	(81.0)	4	(19.0)	0.94	(0.31–2.84)	0.914
Marriage (39 missing)									
Yes	340	(44.2)	271	(79.7)	69	(20.3)	1		
No	429	(55.8)	344	(80.2)	85	(19.8)	0.97	(0.68–1.38)	0.869
Homeless									
No	771	(95.4)	616	(79.9)	154	(20.0)	1		
Yes	37	(4.6)	28	(75.7)	9	(24.3)	1.28	(0.59–2.78)	0.523

**Table 2:** Clinical characteristics among HIV-associated TB patients registered in Tashkent city, Uzbekistan (2013–2017) and their association with TB treatment outcomes.

Characteristics	Total		Favorable Treatment Outcome		Unfavorable Treatment Outcome		Unadjusted OR	95%CI	*p*-Value
	N	(c%)	N	(r%)	N	(r%)			
**Tuberculosis**									
Previous TB treatment (2 missing)									
New	670	(83.1)	546	(81.5)	124	(18.5)	1		
Previously treated	136	(16.9)	98	(72.1)	37	(27.2)	**1.66**	**(1.08–2.54)**	**0.019**
Site of disease (2 missing)									
Pulmonary	659	(81.8)	539	(81.8)	120	(18.2)	1		
Extrapulmonary	122	(15.1)	87	(71.3)	34	(27.9)	**1.76**	**(1.13–2.73)**	**0.013**
Combined	25	(3.1)	18	(72.0)	7	(28.0)	1.75	(0.71–4.28)	0.222
Rifampicin-resistance (101 missing)									
No	499	(70.6)	407	(81.6)	92	(18.4)	1		
Yes	208	(29.4)	169	(81.3)	39	(18.7)	0.98	(0.64–1.48)	0.922
Sputum smear (22 missing)									
Negative	581	(73.9)	480	(82.6)	100	(17.2)	**1**		
Positive	205	(26.1)	156	(76.1)	49	(23.9)	**1.51**	**(1.02–2.22)**	**0.037**
TB diagnosis									
Clinical	185	(22.9)	133	(71.9)	52	(28.1)	**1**		
Bacteriological	623	(77.1)	511	(82.0)	111	(17.8)	**0.39**	**(0.38–0.83)**	**0.002**
Treatment regimen (5 missing)									
Treatment with FLD	585	(72.9)	467	(79.8)	117	(20.0)	1		
Treatment with SLD	218	(27.1)	173	(79.4)	45	(20.6)	1.04	(0.71–1.53)	0.849
Complication of clinical diagnosis (2 missing)									
No	181	(22.5)	151	(83.4)	29	(16.0)	1		
Yes	625	(77.5)	492	(78.7)	133	(21.3)	1.4	(0.91–2.19)	0.129
**HIV**									
ART at admission (150 missing)									
Yes	177	(26.9)	143	(80.8)	34	(19.2)	1		
No	481	(73.1)	389	(80.9)	92	(19.1)	1.0	(064–1.56)	0.981
**Comorbidities**									
Presence of mycotic infection of respiratory tract (343 missing)									
No	259	(55.7)	217	(83.8)	44	(17.0)	1		
Yes	206	(44.3)	172	(83.5)	34	(16.5)	0.96	(0.59–1.57)	0.875
Presence of any other infection (25 missing)									
No	588	(75.1)	477	(81.1)	110	(18.7)	1		
Yes	195	(24.9)	148	(75.9)	47	(24.1)	1.38	(0.93–2.03)	0.106
Diabetes									
No	793	(98.1)	637	(80.3)	155	(19.5)	**1**		
Yes	15	(1.9)	7	(46.7)	8	(53.3)	**4.7**	**(1.68–13.1)**	**0.003**
Hepatitis C (6 missing)									
No	563	(70.2)	462	(82.1)	96	(17.1)	**1**		
Yes	239	(29.8)	176	(73.6)	61	(25.5)	**1.66**	**(1.16–2.40)**	**0.006**
Fluconazole preventive treatment (43 missing)									
No	662	(86.5)	531	(80.2)	131	(19.8)	1		
Yes	103	(13.5)	83	(80.6)	20	(19.4)	0.98	(0.58–1.65)	0.93

TB = tuberculosis; ART = antiretroviral therapy, FLD = First-line drugs, SLD = Second-line drugs. The number in bold demonstrated significant associations.

**Table 3:** Adjusted risk factors associated with unfavorable (failure, lost to follow up, death, not evaluated) treatment outcomes among HIV-associated TB patients registered in Tashkent city, Uzbekistan (2013–2017).

Characteristics	Adjusted OR	95%CI	*p*-Value
Age (every 10-year increase)	1	(0.79–1.26)	0.982
Gender			
Male	1		
Female	0.78	(0.49–1.26)	0.322
Site of disease			
Pulmonary	1		
Extrapulmonary	**2.21**	**(1.38–3.53)**	**0.001**
Combined	2.04	(0.78–5.36)	0.144
Sputum smear			
Negative	1		
Positive	**1.62**	**(1.07–2.40)**	**0.021**
Diabetes			
No	1		
Yes	**5.16**	**(1.77–14.98)**	**0.003**
Hepatitis C			
No	1		
Yes	**1.68**	**(1.14–2.46)**	**0.009**

The number in bold demonstrated significant associations.

## Data Availability

The data that support the findings of this study are available from the corresponding author, S.M., upon reasonable request.

## Open Access Statement

In accordance with WHO’s open-access publication policy for all work funded by WHO or authored/co-authored by WHO staff members, the WHO retains the copyright of this publication through a Creative Commons Attribution IGO license (http://creativecommons.org/licenses/by/3.0/igo/legalcode, accessed on 22 March 2021) which permits unrestricted use, distribution and reproduction in any medium provided the original work is properly cited.
